# Predicting capsule fill weight from in-situ powder density measurements using terahertz reflection technology

**DOI:** 10.1016/j.ijpx.2018.100004

**Published:** 2019-01-04

**Authors:** Sandra Stranzinger, Eva Faulhammer, Jingyi Li, Runqiao Dong, J. Axel Zeitler, Stefano Biserni, Vittorio Calzolari, Johannes G. Khinast, Daniel Markl

**Affiliations:** a Research Center Pharmaceutical Engineering (RCPE), Inffeldgasse 13, 8010 Graz, Austria; b Graz University of Technology, Institute for Process and Particle Engineering, Inffeldgasse 13, 8010 Graz, Austria; c Department of Chemical Engineering and Biotechnology, University of Cambridge, Philippa Fawcett Drive, CB3 0AS Cambridge, UK; d MG2, Via del Savena, 18. I-40065 Pian di Macina di Pianoro, Bologna, Italy; e Strathclyde Institute of Pharmacy and Biomedical Sciences, University of Strathclyde, 161 Cathedral Street, G4 0RE Glasgow, UK; f EPSRC Centre for Innovative Manufacturing in Continuous Manufacturing and Crystallisation, University of Strathclyde, 99 George Street, G1 1RD Glasgow, UK

**Keywords:** Powder bulk density, Terahertz technology, PAT, Capsule filling process

## Abstract

The manufacturing of the majority of solid oral dosage forms is based on the densification of powder. A good understanding of the powder behavior is therefore essential to assure high quality drug products. This is particularly relevant for the capsule filling process, where the powder bulk density plays an important role in controlling the fill weight and weight variability of the final product. In this study we present a novel approach to quantitatively measure bulk density variations in a rotating container by means of terahertz reflection technology. The terahertz reflection probe was used to measure the powder density using an experimental setup that mimics a lab-scale capsule filling machine including a static sampling tool. Three different grades of α-lactose monohydrate excipients specially designed for inhalation application were systematically investigated at five compression stages. Relative densities predicted from terahertz reflection measurements were correlated to off-line weight measurements of the collected filled capsules. The predictions and the measured weights of the powder in the capsules were in excellent agreement, where the relative density measurements of Lactohale 200 showed the strongest correlation with the respective fill weight ($R^{2}=0.995$). We also studied how the density uniformity of the powder bed was impacted by the dosing process and the subsequent filling of the holes (with excipient powder), which were introduced in the powder bed after the dosing step. Even though the holes seemed to be filled with new powder (by visual inspection), the relative density in these specific segments were found to clearly differ from the undisturbed powder bed state prior to dosing. The results demonstrate that it is feasible to analyze powder density variations in a rotating container by means of terahertz reflection measurements and to predict the fill weight of collected capsules.

## Graphical abstract

**Figure:**
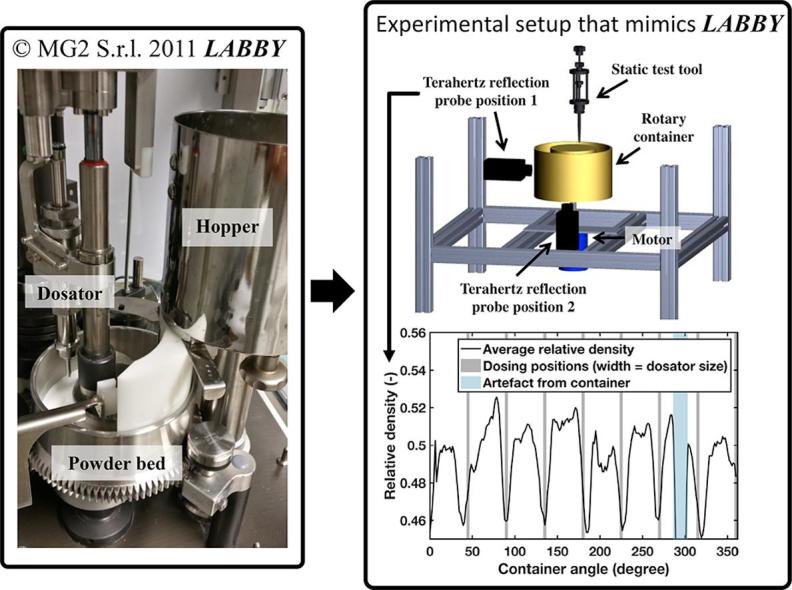

## 1 Introduction

Powder bulk properties are known to be crucial for the operation of many pharmaceutical processes and the quality of final products, such as tablets and capsules. In tablet production, powder density variations may impact tablet mass and hardness, as well as the dissolution performance, hence affecting patients (Singh et al., 2015). Capsules are typically filled with powder or pellets. First of all, filling processes are typically volumetric, i.e., a certain volume of powder is filled into capsules. Thus, only if the density remains constant throughout the process the same mass is filled in every capsule. Second, the fill weight is considered to be directly related to the drug content. Since this requires a uniform blend, both the fill weight variability and content uniformity are critical quality attributes (CQAs) as defined within the Quality-by-Design (QbD) framework (Faulhammer et al., 2014b).

The ability to monitor the powder density during processing of pharmaceutical solids thus would be an enormous benefit, guaranteeing high quality end products. Powder density is also used as a key parameter for the design, optimization, and scale-up of many manufacturing processes by using it as an equipment-independent scaling parameter (Hancock et al., 2003). Furthermore, as the pharmaceutical industry moves from batch towards continuous processing (Plumb, 2005, Buchholz, 2010, Subramanian, 2014), with solid oral dosage forms as one of the primary candidates (Poechlauer et al., 2012), real-time quality monitoring of in-process material is required (Vanarase et al., 2010), possibly leading to real-time release (RTR). Powder density measurements could provide important information in synergy with the process analytical technology (PAT) initiative (FDA, 2004) to enhance end product quality (Singh et al., 2015). However, in-line monitoring of powder density still is an unsolved challenge due to two main reasons:

(1) The powder density is dependent on the type of measurement and the history of processing (Hancock et al., 2003). There are two basic methods of evaluating the density of a powder, the aerated powder (bulk) density and the tapped density (random dense packing). The aerated powder density is determined by allowing the dispersed powder to settle in a container under the influence of gravity. This is often called bulk density. The tapped density is obtained by tapping a container filled with a sample which forces the particles to rearrange leading to a higher density value than the bulk density (Abdullah and Geldart, 1999). This type of analysis provides accurate values for density but can only be performed off-line.
(2) The handling of particulate materials and segregation that may occur since blends consist of aggregates of different particle size (Hastie, 2015) pose a challenge in the development of an in-line powder density monitoring method. In addition, processing effects such as compressive stresses at different levels of the feeders, shear stresses during blending and particle size distribution affect powder density (Frake et al., 1998, Freeman, 2007, Fu et al., 2012, Jallo et al., 2012).

The most common process analyzer in the manufacturing of solid dosage forms is near-infrared spectroscopy (NIRS), which has been extensively described in the literature for in-line monitoring of blend uniformity (Reich, 2005, El-Hagrasy and Drennen, 2006, Li et al., 2006, Moes et al., 2008, Corredor et al., 2014, Li and Qu, 2014, Lin et al., 2015). Other non-invasive techniques, such as Raman spectroscopy (Hausman et al., 2005, Wikström et al., 2006, Arruabarrena et al., 2014), light induced fluorescence (Karumanchi et al., 2011), and chemical imaging (El-Hagrasy et al., 2001, Lyon et al., 2002, Ma and Anderson, 2008, Osorio et al., 2014) are also commonly used for determining the uniformity of a blend in an on-line or in-line setting (Wang et al., 2017). However, even when blend uniformity is adequate, density changes during the process will cause undesirable variations in capsule fill weight leading either to an excess or lack of the active pharmaceutical ingredient (API). It is thus crucial to integrate process analyzers that are capable of controlling the drug concentration and powder density. To date, only a few studies have demonstrated the monitoring of density variations of powder beds. For example, Singh et al. (2015) proposed a method for real-time monitoring of powder bulk density based on NIRS. They also conducted a sensitivity analysis to quantify the effects of powder bulk density on critical quality attributes of pharmaceutical tablets. Recently, NIRS calibration models for real-time prediction of powder density (tap, bulk and consolidated) were developed for a pharmaceutical formulation (Román-Ospino et al., 2016). Other examples for measuring powder density are ultrasound technology (Leskinen et al., 2010), air-coupled acoustic technique (Akseli et al., 2010), photo-acoustic testing (Ketolainen et al., 1995), acoustic emission measurements (Hakanen and Laine, 1995), microwave measurements (Trabelsi et al., 1998), X-ray based methods (Akseli et al., 2011), thermal effusivity monitoring (Ghorab et al., 2007), and electrical tomography (Davies et al., 2005).

A very promising method to study the density variations of particulate material is terahertz technology. The terahertz spectral region covers the frequency range from 0.1 to 4 THz corresponding to wavelengths of 3 mm–30 µm, respectively (Bründermann et al., 2012). Compared with NIR, mid-IR, and Raman spectroscopy, Terahertz time-domain spectroscopy (THz-TDS) is inherently less vulnerable to scattering effects due to its longer wavelength and it is, therefore, a very attractive technique to characterize porous materials, in particular given that most commonly used excipients for the formulation of solid dosage forms are transparent, or semi-transparent, to terahertz radiation. Hence, the pulse of terahertz light can penetrate into and through a specimen leading to a large representative sample volume that is probed for each individual measurement. The penetration depth of the terahertz radiation into the sample material is dependent on the optical properties of the material and the power of the terahertz pulse. At present, penetration depths into typical pharmaceutical formulations have been demonstrated up to 5.3 mm (Markl et al., 2017a).

The terahertz technology can also be employed to measure the effective refractive index of a sample in a non-destructive manner (Bawuah et al., 2016, Markl et al., 2017a, Markl et al., 2017b). The effective refractive index is a function of the fill fractions of each constituent and the total porosity. It can therefore be used to determine the porosity of a tablet within seconds by knowing the other fill fractions (e.g., knowing the formulation). Recently, we have demonstrated that powder density variations in a rotating container can be captured and analyzed by means of terahertz pulsed imaging (Stranzinger et al., 2018a). This setup and methodology were capable of spatially resolving relative density variations as small as 0.3% (Lactohale 100) in a moving container.

In the present study we applied this new terahertz-based method to analyze the correlation of the measured powder bed density with the fill weight in a capsule. This was performed by drawing small quantities from the powder bed using a novel sampling system that mimics the capsule filling process (Stranzinger et al., 2018b) in proximity to the rotating container. Hence, the relative density of the powder bed was monitored and correlated with off-line weight measurements to predict the fill weight based on the terahertz reflection measurement. We further analyzed the effect of sampling on the powder bed density for the different materials and two different terahertz probe positions, i.e. from the side (terahertz probe position 1) and the bottom (terahertz probe position 2) of the rotary container.

## 2 Materials and methods

### 2.1 Materials

Three different grades of α-lactose monohydrate excipients were used as received from the supplier (DFE pharma, Goch, Germany). These grades are commonly used as carriers in inhalation therapies. Sieved Lactohale 100 and two milled powders, Lactohale 200 and Lactohale 220, were selected on the basis of previous research efforts of our group focusing on their processability in a low-dose capsule filling process (Stranzinger et al., 2017, Stranzinger et al., 2018b). In these studies a comprehensive bulk powder profiling was performed that aimed to understand the effect of material attributes on the capsule filling performance. Table 1 summarizes the most relevant powder properties. The height of the powder layer in the container was kept constant at 10 mm by adjusting the mass of the powder (see Table 1). According to the flow function coefficient and the cohesion values, Lactohale 100 has the best flowability, followed by Lactohale 200 and Lactohale 220, which exhibits poor flowability (Stranzinger et al. 2017). As reported in our previous work (Stranzinger et al., 2018b), the Hausner ratio and the Angle of Repose, two alternative powder flowability indices followed the same trend for these lactose powders.

**Table 1:** Properties of the various lactose excipients used in this study. The mass in this table refers to the theoretical weight of the powder filled in the container (calculated based on the bulk density of the respective powder).

Material	Bulk density (g cm^−3^)	Tapped density (g cm^−3^)	True density (g cm^−3^)	Particle size x50 (µm)	Flow function coefficient	Cohesion (kPa)	Mass (g)
Lactohale 100	0.697 ± 0.004	0.828 ± 0.013	1.539 ± 0.003	155.2 ± 0.6	6.58 ± 0.01	0.24 ± 0.02	70.9
Lactohale 200	0.622 ± 0.003	0.996 ± 0.002	1.543 ± 0.002	78.7 ± 0.4	4.04 ± 0.01	0.39 ± 0.01	63.3
Lactohale 220	0.400 ± 0.007	0.785 ± 0.007	1.547 ± 0.004	13.4 ± 0.0	1.65 ± 0.01	1.05 ± 0.13	40.7

### 2.2 Experimental setup

**Fig. 1:**
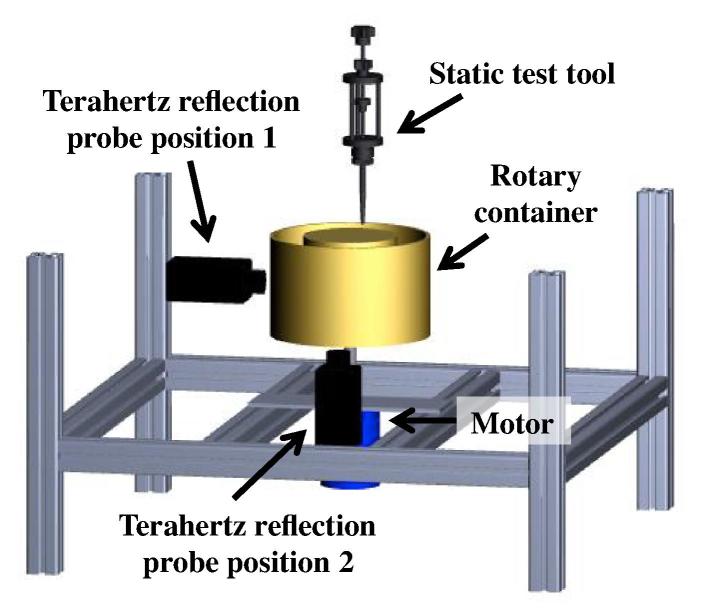
Schematic of the experimental setup for terahertz reflection probe position 1 and 2.

A schematic of the experimental setup, comprising the rotary container, the terahertz fiber-based flexible reflection probe (at position 1 and 2) and the static test tool, is depicted in Fig. 1. The rotary container is made of high-density polyethylene (HDPE), which is transparent to terahertz radiation. The height of the interior of the container is 45 mm. A metal ring with a thickness of 10 mm combined with a load sensor block of 63 mm thickness, both components later referred to as the compression unit, were used to apply pressure on the powder bed and thus causing a change of its relative density. The pressure on the powder bed was adjusted by tightening a nut on the screw to increase the compaction pressure on the powder bed. Three load sensors connected to a computer via an Arduino Uno (Arduino, Somerville, US) were used to measure the applied force, which was only measured prior to rotation at each compaction stage. For details about the force sensors and installation we refer to our previously published work (Stranzinger et al., 2018a).

**Fig. 2:**
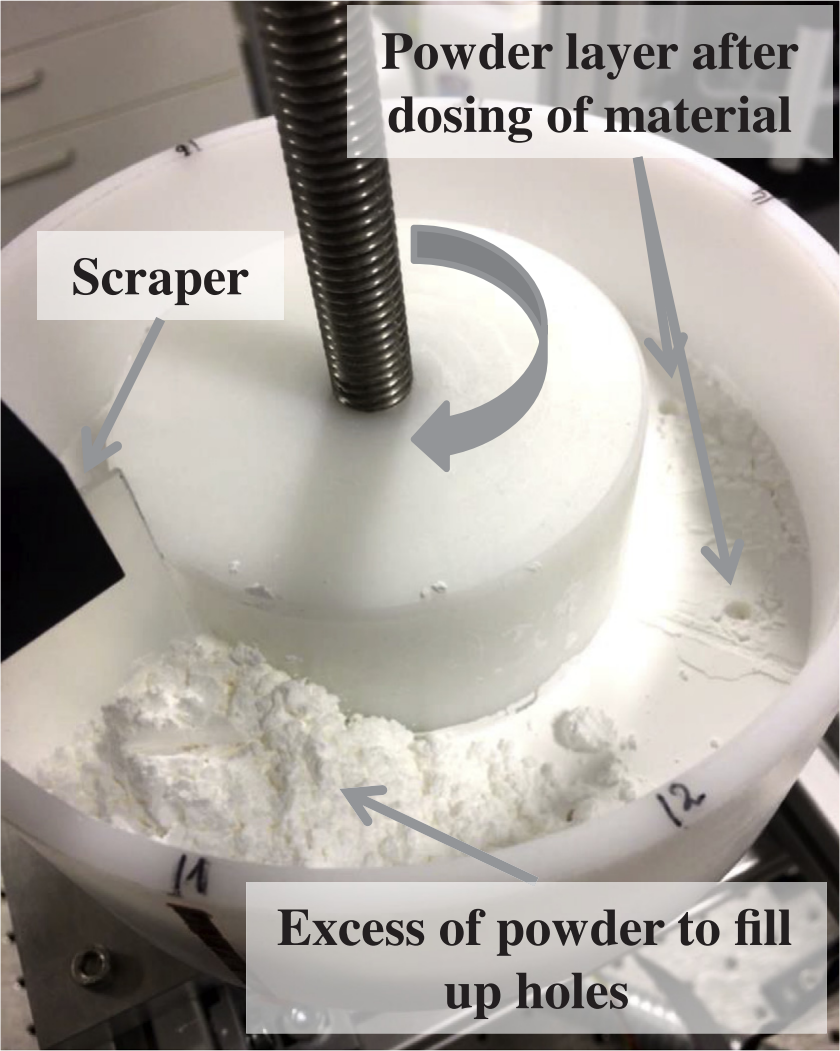
Experimental setup for simulating a conventional dosator nozzle capsule filling process.

The experimental setup was developed to closely mimic a conventional dosator nozzle capsule filling process (Fig. 2). By rotating the container in clockwise direction, the holes which are introduced in the powder bed after each dosing step are filled with excess powder that is placed immediately behind the scraper. Several rotation cycles are typically needed to completely fill the holes in the powder layer using this setup. To date, the filling of the holes has only been investigated visually from the top of the powder layer. Here, terahertz reflection measurements through the powder bed at two further positions were performed to gain more insights into the powder bed state after dosing.

### 2.3 Powder bed preparation

For each experimental run the required amount of powder (as shown in Table 1) was manually filled into the rotary container. A scraper was used to smoothen the powder layer prior to measuring the powder layer height and subsequently mounting the compression unit. The pressure on the powder bed was adjusted by displacing the compression unit. This displacement was measured manually at eight evenly spaced positions from the bottom of the container to the top of the compression block using a digital caliper prior to the start of every measurement. An average value of this displacement was then used to calculate the fill height, $\overset{-}{h}$, and eventually to determine $\overset{-}{\varrho }$_r_ (Eq. (1)). Moreover, for each compaction step the applied pressure on the powder bed was measured using the integrated force sensors (see Section 2.2). The motor speed of 4 rpm was kept constant throughout all experiments. For each experiment the average relative density, ${\overset{-}{\varrho }}_{\text{r}}$, was calculated by

(1) $${\overset{-}{\varrho }}_{\text{r}}=\frac{m}{V\varrho_{\text{t}}}=\frac{m}{A\overset{-}{h}\varrho_{\text{t}}},$$

where A is the cross-section area of the container, m is the powder mass and $\varrho_{t}$ is the true density (both shown in Table 1). A was constant throughout all measurements and is given as

(2) $$A={\left(\frac{D_{o}-D_{i}}{2}\right)}^{2}\pi$$

with $D_{o}=138$ mm and $D_{\text{i}}=78$ mm as the outer and inner diameter of the interior of the container.

### 2.4 Terahertz reflection measurements and prediction of relative density variations

Terahertz reflection measurements were continuously acquired for three independent experimental runs per material, and the average relative densities of the five different compaction steps in each run were predicted. A commercial time-domain terahertz system (TeraPulse 4000, TeraView Ltd, Cambridge, UK) coupled with a fiber-based flexible reflection probe was used in this study. The system was configured to operate at an optical time delay of 45 ps and an acquisition rate of 15 Hz. The reflection probe was equipped with a silicon lens with a focal length of 18 mm. The beam waist at the focus was ≈600 µm. 2000 terahertz waveforms were acquired continuously for each compaction step, which yielded a total measurement time of 133 s. These 2000 measurements covered about 8.5 rotations of the container. A thin strip of copper foil was attached onto the outside of the container, which acted as a datum as it reflected the terahertz pulse and facilitated the automatic detection of a full rotation from the terahertz waveforms. Seven full rotations were averaged and yielded in N=230 terahertz waveforms uniformly distributed around the container.

The refractive index of the powder, $n_{p}$, was calculated by measuring the reflection coefficient, $r_{\text{cp}}$, (from container and powder) and utilizing its relationship which is given as

(3) $$r_{cp}=\frac{n_{c}-n_{p}}{n_{c}+n_{p}},$$

where $n_{c}=1.54$ (refractive index of the HDPE at terahertz frequencies (Wietzke et al., 2011)) is the refractive index of the container. Eq. (3) can then be rearranged to

(4) $$n_{p}=\frac{n_{c}\left(r_{\mathrm{cp}}+1\right)}{n_{c}-r_{\mathrm{cp}}}.$$

$r_{\mathrm{cp}}$ was calculated by relating the amplitude of the reflected pulse from the container/powder interface to a reflection from the copper foil on the container. This reference reflection from the copper foil was acquired prior to every experiment.

A linear relationship between the measured refractive indices and the relative densities of the powder in the container was observed (for details see Stranzinger et al. (2018a)), and therefore a linear model was fitted to the calibration data. In particular, the model is expressed as

(5) $${\overset{-}{\varrho }}_{r}=a_{0}+a_{1}{\overset{-}{n}}_{p}$$

with $a_{0}$ and $a_{1}$ as fitting parameters. ${\overset{-}{\varrho }}_{\text{r}}$ is the average relative density from Eq. (1) and ${\overset{-}{n}}_{p}=\sum_{i=1}^{N}n_{p,i}$ is the average refractive index with $n_{p,i}$ calculated by Eq. (4) and the terahertz reflection measurement at position i.

The noise in the terahertz data was reduced by applying a moving average filter with a size of 5 to the data: the reflection coefficient of 5 successive terahertz time-domain waveforms were averaged.

### 2.5 Static mode tests for off-line weight measurements

**Fig. 3:**
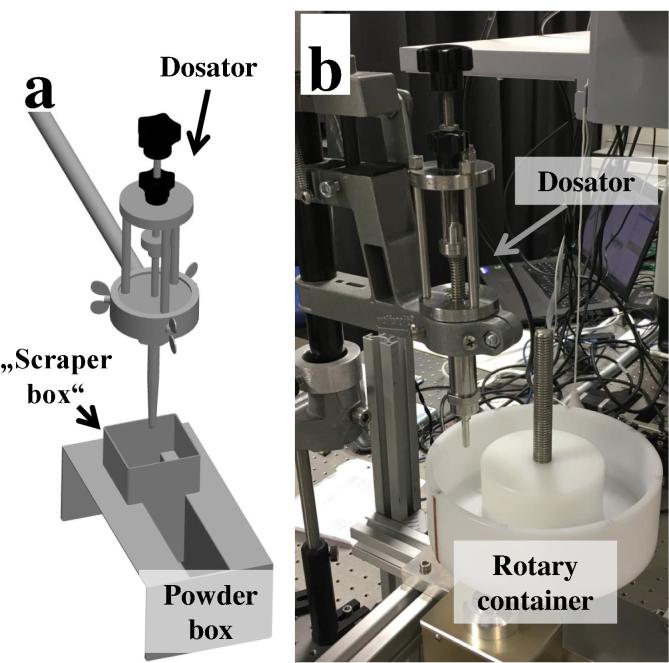
(a) Schematic of the stand-alone static test tool including a rectangle powder box. (b) The static test tool setup used in the present study, i.e. the tool is placed next to the rotary container filled with powder.

**Fig. 4:**
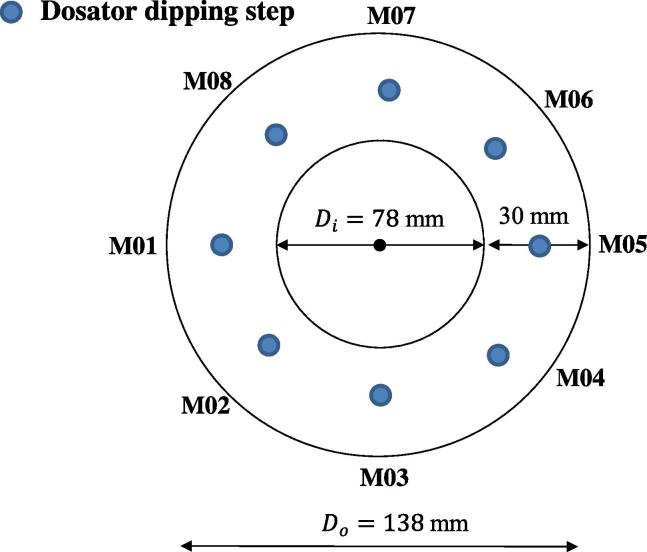
Schematic of the rotary container dimensions and the eight off-line sampling positions (M01–M08).

Off-line weight measurements of powder samples were performed on samples drawn from the rotary container by a novel test setup, i.e., a stand-alone static test tool (Fig. 3a), which was previously introduced by our research group (Stranzinger et al., 2018b). Essentially, the static test tool takes samples via a dosator head from a static powder bed, mimicking the dosing step in a dosator capsule filler. As shown in Fig. 3b, the static test tool was placed next to the rotary container. Dosator dipping steps, i.e., downward movement of the dosator, collecting of powder from the rotary container and ejection of powder into a capsule were performed using the following process parameters: a dosing chamber length of 5 mm, a dosator diameter of 3.4 mm and an initial powder layer height of 10 mm. This resulted in a pre-compression ratio (i.e., the ratio between the dosing chamber length and the powder layer height) of 1:2. Dosator dipping steps were performed at the eight different positions shown in Fig. 4. The collected capsules were stored in cups sealed with Parafilm until their fill weight and weight variability were analyzed (as described in Section 2.6).

Fig. 4 depicts the eight different positions around the rotary container (M01–M08) from where powder was sampled for off-line weight measurements. For each material and compression step the fill weight of eight samples was averaged.

### 2.6 Analysis of capsule fill weight and weight variability

The collected samples were weighed on a Denver (SI-234A) analytical scale. Due to a relatively high weight of empty capsules and their variability, the collected filled capsules were weighed, emptied and re-weighed. Compressed air was used to clean any residual powder from the capsule surfaces in order to ensure that no powder was left in the capsule after it was emptied.

## 3 Results and discussion

### 3.1 Correlation between in-line predicted relative densities and off-line weight measurements

Firstly, a linear model between the measured refractive index and the nominal relative density was developed. We observed an excellent linear relationship between the measured refractive index and the relative density for each powder, as reflected by the high correlation coefficient ($R^{2}$) and small root mean square error (RMSE) shown in Table 2. One linear model was developed per material, which was then used to predict the relative densities for all three experiments per material.

**Table 2:** Correlation coefficient and root mean square error (RMSE) for the linear model (Eq. (5)) relating the measured refractive index to the relative density for the three different powders.

Material	$R^{2}$	RMSE
Lactohale 100	0.982	0.002
Lactohale 200	0.996	0.003
Lactohale 220	0.995	0.003

We investigated the accuracy of the terahertz-based density measurements for Lactohale 100 in our previous study (Stranzinger et al., 2018a). The measurement accuracy of the reflection coefficient is approximately 0.0015, which results in an accuracy of 0.3% for the relative density.

**Fig. 5:**
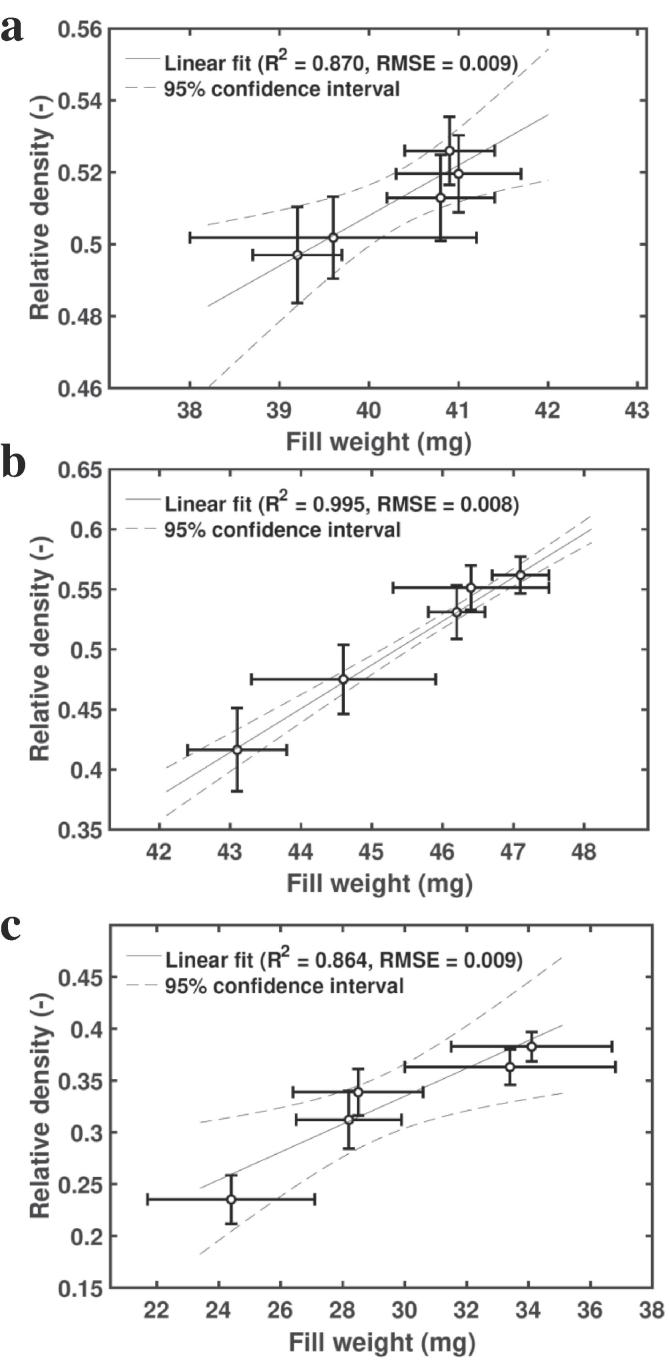
Comparison of the relative density from in-line terahertz reflection measurements and off-line weight measurements. (a) Lactohale 100, (b) Lactohale 200 and (c) Lactohale 220. The relative density predictions were averaged for three experiments per material. The fill weight is the average of eight samples per material and compression step.

The predicted relative densities of all three experiments were averaged and then correlated with the off-line weight measurements (Fig. 5). We observed the highest correlation coefficient ($R^{2}=0.995$) for Lactohale 200, which emphasizes that in-line measured relative bulk densities are highly suitable to predict the change in fill weights of off-line samples. A significant increase in fill weights from 43.1 to 47.1 mg was observed for this powder. This can be correlated with the compressibility of the powder as previously discussed by Stranzinger et al. (2017), where a compressibility of 12.66% for Lactohale 200 was reported at a compaction pressure of 8 kPa. In terms of fill weight variability, very low relative standard deviations (<3%) were achieved for this powder, which is highly important in order to guarantee a high quality product during capsule filling.

For Lactohale 100 a correlation coefficient of $R^{2}=0.870$ was determined. Given the limited compressibility (1.05% at 8 kPa (Stranzinger et al., 2017)) it was not possible to significantly vary the relative density of Lactohale 100 using the experimental setup. The setup would need a significant modification in order to make it suitable to achieve higher normal forces during compaction. However, it is important to highlight that such high forces are not expected to be relevant for typical capsule filling processes.

**Fig. 6:**
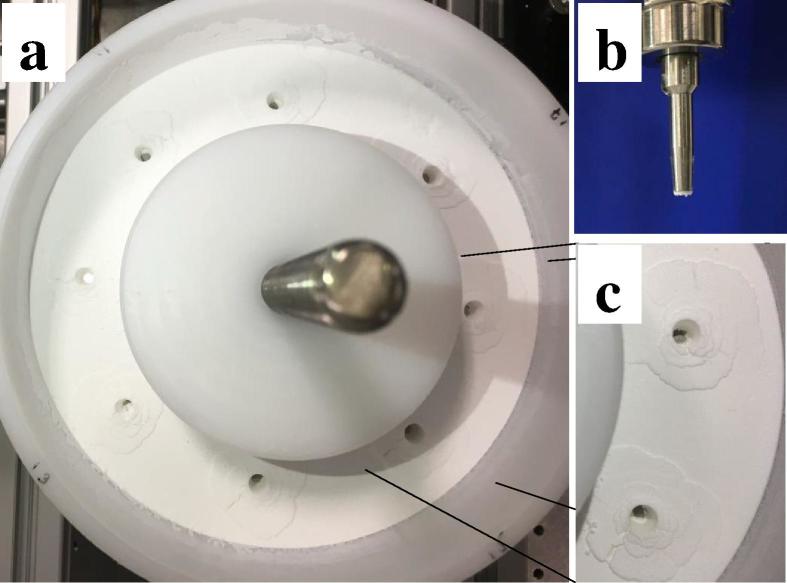
Experiments with Lactohale 220. (a) Powder bed after dosing of material. (b) Dosator with the collected powder after the dosator dipping step. (c) Holes in the powder layer after dosing of material.

A correlation coefficient of $R^{2}=0.864$ was observed for the highly cohesive Lactohale 220 powder. The fill weights significantly increased from 24.4 to 34.1 mg as a function of applied normal forces, owing to the high compressibility of this grade of lactose (36.95% at 8 kPa (Stranzinger et al., 2017)). As expected, the highly cohesive powder yields the highest fill weight variability amongst the three powders. These results are in line with the previously reported weight variability of this excipient (Stranzinger et al., 2017, Stranzinger et al., 2018b). In a later study, Stranzinger et al. (2018b) detected a higher weight variability for this highly cohesive powder when sampled without vibration, which was the mode of sampling strategy in the present study. Another interesting discovery in terms of weight variability of this grade of excipient was observed by visually examining the powder bed after dosing (Fig. 6a and c): It can be clearly seen that in some cases the dosator collected more powder, i.e., the bottom of the container is visible, whereas in other cases some powder remained in the container. This phenomenon is expected to be relevant with regards to the fill weight variability of collected capsules. An important fact to be considered for this type of powder is that the fill weight for highly cohesive powders is also affected by frictional characteristics (wall friction angle) (Faulhammer et al., 2014b). For highly cohesive powders it can be expected that a combination of friction together with greater inter-particle forces is sufficient to keep them inside the nozzle. However, as reported by Stranzinger et al. (2018b), a pre-compression ratio of 1:2 would require a smaller gap (i.e., the difference between the lowest point of the dosator and the bottom of the container) than the gap used in the present study. A smaller gap could provide the critical compression of the powder required for a low variation inside the dosator. In other words, it is highly relevant to also consider the gap as emphasized by the previous work from Stranzinger et al. (2018b) in addition to critical process parameters investigated in earlier studies (Faulhammer et al., 2014a, Faulhammer et al., 2014b, Stranzinger et al., 2017).

### 3.2 Effect of dosing of material on powder bed state

**Fig. 7:**
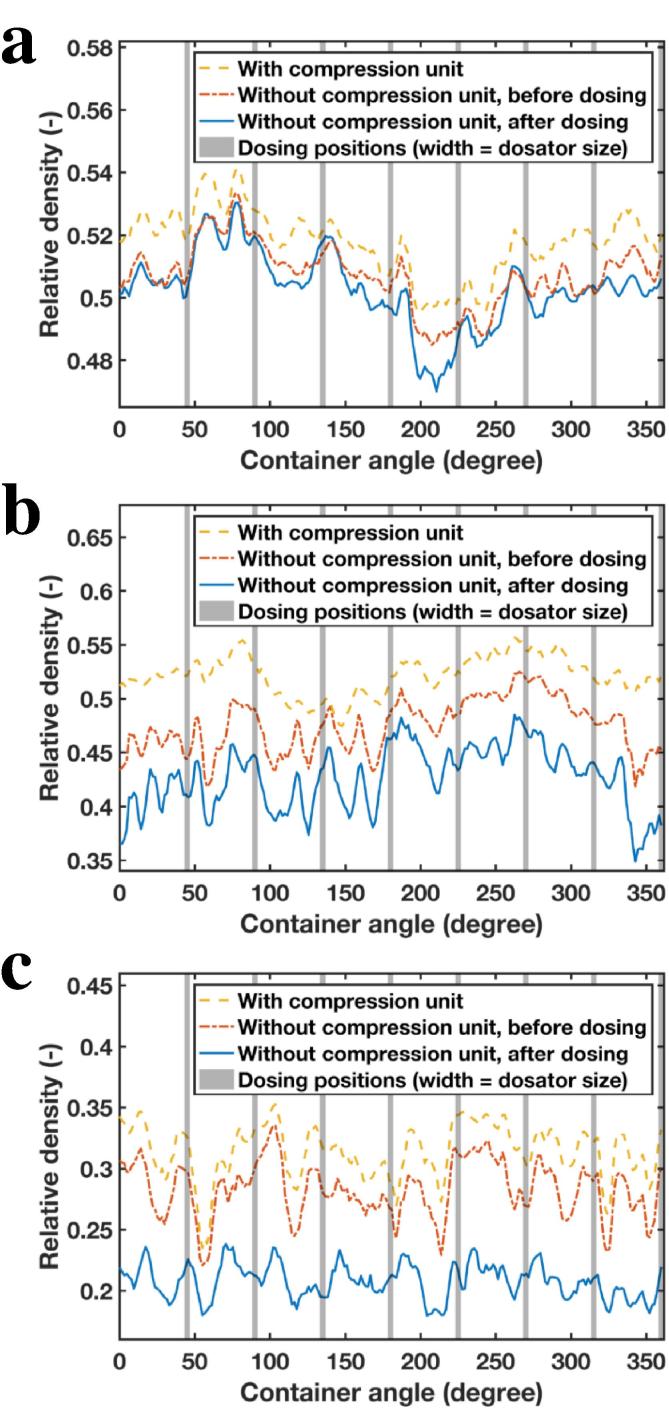
Impact of dosing of material on the powder bed state. The relative densities of the powder bed as a function of the container angle are shown with the compression unit (yellow dashed line), without the compression unit (red dash-dot line) and after dosing (blue solid line). (a) Lactohale 100, (b) Lactohale 200, (c) Lactohale 220. (For interpretation of the references to colour in this figure legend, the reader is referred to the web version of this article.)

In order to evaluate the impact of dosing of material on the relative density of the powder bed, terahertz reflection measurements (using terahertz probe position 2) were acquired continuously during rotation of the container for three different conditions (1) powder bed with the compression unit for displacement position max (i.e., maximum applied normal force), (2) powder bed after removing the compression unit and before dosing, and (3) powder bed without the compression unit and after dosing of material. As shown in Fig. 7, dosing of material considerably affects the state of the powder bed, but to a different extent for the three materials under investigation. Not surprisingly, the largest range of relative densities was observed for Lactohale 220, i.e. the powder that undergoes the largest volume change when it is compressed. Interestingly, relative densities of Lactohale 100 as a function of the container angle (i.e., local relative densities) varied to a greater extent for the different specific angle positions compared to Lactohale 200 and Lactohale 220, indicating a more non-uniform powder bed upon compaction. This is in line with our previous study (Stranzinger et al., 2018a), where maps of relative density distributions around the container (in horizontal direction) showed a similar trend for this powder. Thus, for such powders the filling process seems to be a crucial part during processing, whereas for powders with characteristics similar to Lactohale 200 and Lactohale 220 the dosing step itself has to be regarded critically, since it greatly impacts the powder bed condition and consequently the quality of the end product, i.e., the filled capsules.

**Fig. 8:**
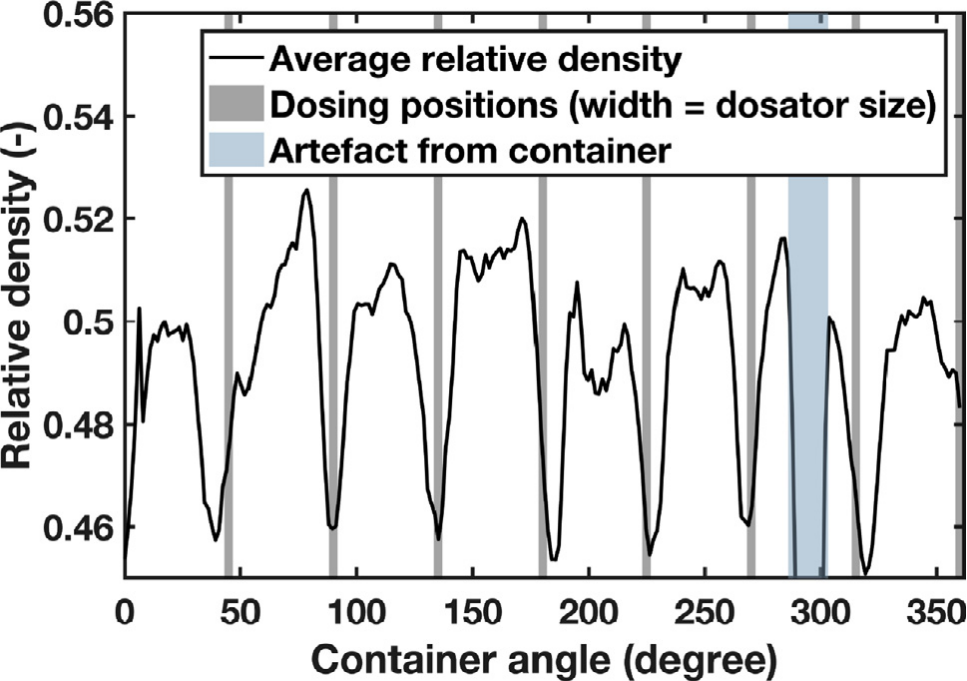
Average relative density of the powder bed after dosing of material as a function of the container angle for Lactohale 100.

**Fig. 9:**
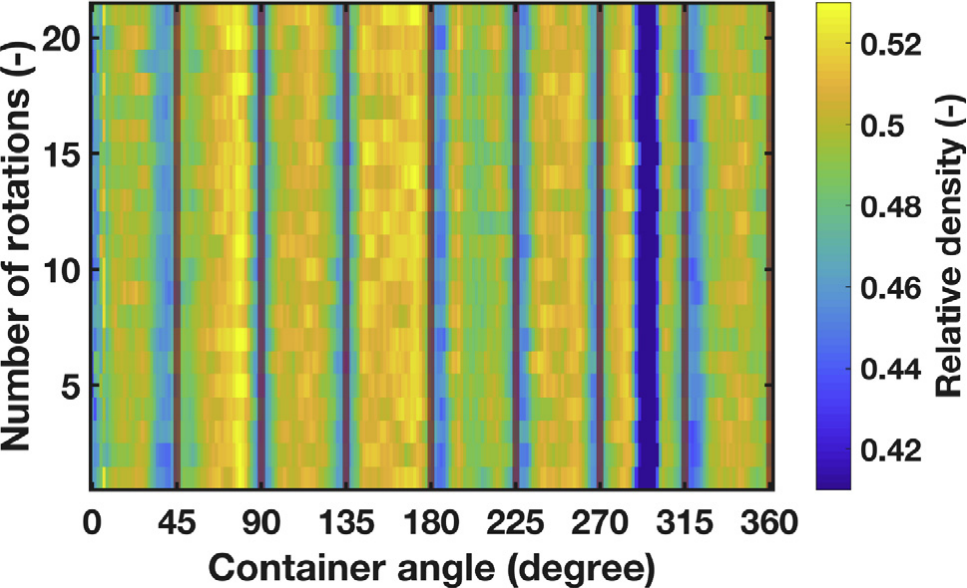
Map of relative density distributions as a function of increasing number of rotation of the container and the angle on the container for Lactohale 100. The dark blue region (very low relative density) at a container angle of about 290° is an artefact from the container. (For interpretation of the references to colour in this figure legend, the reader is referred to the web version of this article.)

As described in Section 2.2 (Fig. 2), the developed method was tested for its applicability in a conventional dosator nozzle capsule filling process. In those experiments after conditioning the powder layer, i.e., having a smooth powder layer of specific powder layer height, eight dosator dipping steps were carried out, followed by rotating the container to fill the introduced holes in the powder bed. Terahertz reflection measurements (using terahertz position 2) were continuously acquired throughout the entire process, i.e., rotating the container for 2 min to fill the holes. The data in Fig. 8, Fig. 9 reveal that at each dosing position the spatially resolved relative densities are notably lower compared to the rest of the powder layer where no samples were taken. Interestingly, these variations of relative densities for segments where powder was collected are visible throughout the filling process (Fig. 9). This finding indicates that even though the holes seemed to be filled (by visual inspection), the relative density in these specific segments differ from the initial powder bed state.

By measuring the actual relative density of those spots of interest in the powder bed, the developed method will improve existing solutions in terms of predicting the target fill weight and weight variability. Moreover, these results encourage the applicability of this method for monitoring powder bulk densities throughout the entire capsule filling process. It is of utmost importance to measure the relative density at sampling positions to detect any deviations from the target relative density of the powder bed. Powders with characteristics, such as small particles, poor flowability, high cohesion, are prone to densify under mechanical vibrations of the powder drum during the filling process, resulting in higher fill weights over time (Stranzinger et al., 2017). This was also discussed in the study by Llusa et al. (2013), where they found a clear correlation between more intense mechanical vibrations during capsule filling and fill weight changes. The information of changes of the powder bulk density from in-situ terahertz reflection measurements could trigger corrective actions such as the adaption of the dosing chamber length, the powder bed height, or even the gap between the lowest point of the dosator at the end of a stroke and the bottom of the powder container. The latter parameter was recently reported by Stranzinger et al. (2018b) to be a critical process parameter for the filling of powder using a dosator nozzle machine for capsule filling.

## 4 Conclusion

We demonstrated the applicability of the terahertz technology as a new process analyzer to determine powder bulk density variations in a moving powder bed. Terahertz reflection technology was employed to measure changes in the bulk density non-destructively throughout the process, which were further correlated to weight measurements of collected off-line samples. The high potential of this technology was evident from the observed correlation coefficients of $R^{2}=0.870$, $R^{2}=0.995$ and $R^{2}=0.864$ for Lactohale 100, Lactohale 200 and Lactohale 220, respectively, between the predicted relative densities from in-line terahertz measurements and off-line weight measurements. It was found that the repacking of voids left by the dosator with loose powder clearly results in a strong heterogeneity of the relative density in the resulting powder bed that remains even after applying a load to the bed. The findings of this study provide a new perspective of the terahertz technology to be used as an in-line monitoring tool for manufacturing processes where powder bed variations are directly linked to the quality of final products, e.g. feeder, rotary container used in a dosator or tamping pin capsule filling process. A critical factor of the presented methodology is the positioning of the terahertz probe relative to the moving container. The powder bed density at the position where the dosator collects the powder could be measured by placing the terahertz probe at the respective position.

The implementation of a process analyzer in capsule filling processes could significantly reduce the fill weight variability in capsules. The knowledge about the state of the powder bed throughout the process will help in designing production machines, e.g., introduction of tools to stabilize the powder bed condition, which are capable to maintain a constant powder bed density and thus reduce process induced variability of final products.

## Declaration of interests

The authors declare that they have no known competing financial interests or personal relationships that could have appeared to influence the work reported in this paper.
